# Investigation of optimum minimum segment width on VMAT plan quality and deliverability: A comprehensive dosimetric and clinical evaluation using DVH analysis

**DOI:** 10.1002/acm2.13417

**Published:** 2021-09-30

**Authors:** AB Mohamed Yoosuf, Muhammad Bilal Ahmad, Salem AlShehri, Abdulrahman Alhadab, Mamdouh Alqathami

**Affiliations:** ^1^ Department of Radiation Oncology Ministry of National Guard—Health Affairs Riyadh Saudi Arabia; ^2^ King Abdullah International Medical Research Center Riyadh Saudi Arabia; ^3^ King Saud bin Abdulaziz University for Health Sciences Riyadh Saudi Arabia

**Keywords:** DVH, IMRT, plan quality, segment width, VMAT

## Abstract

**Purpose:**

Minimum segment width (MSW) plays a fundamental role in the shaping of optimized apertures and creation of segments of varying sizes and shapes in complex radiotherapy treatment plans. The purpose of this work was to study the effect of MSW on dose distribution in patients planned with VMAT for various treatment sites using dose volume histogram (DVH) analysis.

**Materials and methods:**

For the validation of optimum MSW, 125 clinical treatment plans were evaluated. Five groups were identified (brain, head and neck, thorax, pelvis, and extremity), and five cases were chosen from each group. For each case, five plans were created with different MSW (0.5, 0.8, 1.0, 1.25, and 1.5 cm). The quality of treatment plans created using different MSW were compared using dosimetric indicators such as target coverage (*D*_98_—dose to 98% of the planning target volume (PTV), maximum dose (*D*_2_—maximum dose to 2% of the PTV), monitor units (MU), and DVH parameters related to organs at risk (OAR). The effect of the MSW on delivery accuracy was quantitatively analyzed using the measured fluence utilizing ionization chamber‐based transmission detector and model‐based dose verification system. Traditional global gamma analysis (2%, 2 mm) and dose volume information was gathered for the PTV and organs at risk and compared for different MSWs.

**Results:**

A total of 125 plans were created and compared across five groups. In terms of treatment plan quality, the plans using MSW of 0.5 cm was found to be superior in all groups. PTV coverage (*D*_98_) decreased significantly (*p* < 0.05) as the MSW increased. Similarly, the maximum dose (*D*_2_) was found to be increased significantly (*p* < 0.05) as the MSW increased from 0.5 cm, with MSW of 1.5 cm being the least in terms of plan quality for both PTVs and OARs. In terms of plan deliverability using DVH analysis, treatment planning system (TPS) compared to measured fluence, VMAT plans produced with MSW of 0.5 cm showed a better dosimetric index and a smaller deviation for both PTVs and OARs. The deliverability of the plans deteriorated as the MSW increased.

**Conclusion:**

Dose volume histogram (DVH) analysis demonstrated that treatment plans with minimal MSW showed better plan quality and deliverability and provided clinical relevance as compared to gamma index analysis.

## 1 INTRODUCTION

Modern‐day literature suggests that volumetric modulated arc therapy (VMAT) is an effective and safe treatment plan modality for most cancer treatments.^1^, ^2^ The VMAT plan technique has been identified as an excellent approach that enables steep dose gradients, optimum precision, reduced treatment time, for that reason minimizing the chance of intrafraction setup deviations or organ movement, improvement in outcome, and the capability for escalating the dose to the targets whilst lowering acute and late toxicities.^3^, ^4^ However, as for all superior treatment techniques, one essential issue is to ensure the consistency between treatment planning and delivery. This is to preclude the risk of accidental mistreatments with potentially severe implications for patients. For VMAT, the resolution of the fluence can have a direct effect on the quality of the treatment plan, which affects treatment effectiveness in addition to complications due to radiotherapy.^5^, ^6^ Furthermore, in addition to the tradeoff between complexity and plan quality, that is, target dose coverage, normal structure sparing, another tradeoff that should be considered during the treatment planning process is that between complexity and the dosimetric accuracy of the treatment plan.^2^, ^3^

An optimum radiotherapy treatment plan can be thought of as an improved analytical arrangement of the dosimetric and physical constraints that reduce dose to normal structures, decrease hot and cold volumes, and shorten treatment time while maintaining the recommended dose to tumor volume. Consequently, treatment plans can be accomplished in numerous ways with a small distinction in these factors without significantly affecting the quality.^7^ VMAT plans are made up of large number of long, small, and irregular segments based on the plan constraints and PTV volumes. Within the TPS, the minimum segment width (MSW) parameter plays a critical role in the shaping of optimized apertures as well as the creation of segments of varying sizes and shapes. By and large, in treatment planning, intensity distributions formed by optimization methods are altered into multileaf collimator (MLC) leaf trajectories in order to provide preferred dose distributions of any shape.^8^, ^9^

A comprehensive quality assurance (QA) program ensures the effectiveness and safety of radiation therapy.^10^ From a clinical perspective, the QA results used for dose verification should focus on detrimental dose differences, as these will ultimately influence whether or not a treatment plan is justified for treatment.^11^ Under/over dosage in patient‐specific structures may lead to negative outcomes.^12^, ^13^ The gamma index (GI) assessment method, which integrates spatial data and dose differences, has been the standard method for verifying VMAT dose verification so far.^14^, ^15^

Measurements based on GI analysis with different detectors provides useful information about whether linear accelerators are performing as expected, but there is no correlation between clinical metrics and pass rates.^10^, ^16^ In addition, GI analysis is limited in accuracy in areas of steep dose gradients. Further, it is difficult to extrapolate gamma‐ray transmission to clinical outcomes because the GI estimates lack information on doses to patient specific structures. This is supported by studies showing that gamma‐pass rates are weakly correlated with differences in target volume and doses in OARs.^17^, ^18^, ^19^, ^20^, ^21^, ^22^ To overcome these limitations, in addition to gamma transmission, information about DVH can be included in the QA procedure.

Recently, several vendors have developed tools to capture the fluence generated by the delivery system prior to patient treatment in order to determine the definite dose delivered within the patient model using DVH analysis. The Dolphin Compass System (IBA Dosimetry, Schwarzenbrook, Germany) is a commercially available dosimetry solution that can be used to reconstruct 3D doses, based on measured fluence, in computed tomography (CT) images of a phantom or patient.

Compass verification system that comprises ionization chamber‐based transmission detector (Dolphin) mounted on the linear accelerator head can quantify the yield fluence of any random field. This measured fluence can be utilized as an input to calculate 3D dose distributions based on collapsed cone algorithm, either in phantom or patient. The Compass system has been investigated for its inherent accuracy in comparison to other measuring instruments, as well as its clinical usability in the assessment of DVH distribution analysis between planned and measured doses.^22^, ^23^, ^24^, ^25^

Previous studies have reported the optimal MSW in terms of VMAT plan quality using GI analysis for specific treatment sites.^8^, ^26^, ^27^, ^28^ However, as of now, there are no reports of optimal MSW for all sites and their clinical significance on plan deliverability using DVH analysis. The purpose of this work was to study the effect of MSW on dose distribution in patients undergoing VMAT for various treatment sites using DVH analysis, utilizing Compass verification system and Dolphin detector.

## 2 MATERIALS AND METHODS

For the validation of optimum MSW using DVH analysis, 25 clinical treatment plans were selected retrospectively. The aim of the study was to look into a variety of clinical treatment plans designed with VMAT. As a result, five groups were identified, and five patients were chosen from each of them. The brain, head and neck (HN), thorax, pelvis, and extremity were divided into five groups, each representing a standard dose prescription and fractionation schedule with varying target volumes. Patients with varying dose prescriptions were included and analyzed in terms of percent dose for comparison. For each patient, the PTV as well as different OAR were considered. Further, the dose constraints used in the study were based on the QUANTEC and RTOG protocols.^29^, ^30^

### 2.1 Treatment planning

For all patients, the VMAT plans were created using the CMS Monaco (v5.2.11 Elekta, Crawley, UK) treatment planning system (TPS) that utilizes the Monte Carlo algorithm. In Monaco TPS, treatment planning is a two‐step process that begins with the creation of theoretical fluence based entirely on dose constraints, followed by the segmentation of theoretical fluence into deliverable MLC segments.^9^ Treatment plans were created using either single arc (*n* = 9) or two arcs (*n* = 16) based on patient anatomy. The maximum number of control points per arc was set to 150 with a calculation grid resolution of 2.5 mm and a statistical uncertainty of 1% per calculation.

#### 2.1.1 Minimum segment width

In Monaco, the workflow is based on an alternative sliding window model in which all MLC leaves move in a constant unidirectional motion from the start position to the end position. The leaf assembly moves in one direction first, then the other, alternating the sectors of the complete arc and changing the speed of the leaf, creating spaces between opposing leaves while the system modulates the intensity of the delivered fluence. The Minimum Segment Width (MSW) parameter was used in the sequencing algorithm to determine the minimum leaf distance between two opposing leaves in the segmented field. As a result, the MSW parameter was generated to produce a narrow segment sequence with a limited number of segments for scheduled delivery. The MSWs valid range is 0.5–2.0 cm. The recommended MSW, as per the vendor, is 0.5 cm.^31^ For each case in the study, five VMAT plans were created with different MSWs of 0.5, 0.8, 1.0, 1.25, and 1.5 cm. For all plans with different minimum segment widths, physical parameters like number of arcs, arc length, increment, fluence smoothing (Medium), and cost functions for the plan optimization were kept unchanged as our goal was to study the impact of minimum segment width on plan quality and deliverability.

#### 2.1.2 Treatment plan quality

Each patient was planned based on ICRU 83 with a goal of delivering 98% of the prescribed dose to at least 95% of the tumor volume and allowing a maximum dose of up to 107%.^20^ For OARs, the maximum dose for serial organs and volume restrictions for parallel structures were observed as a function of total dose and dose per fraction using RTOG protocols.^20^, ^29^ The different MSW plans have been compared to dosimetric indicators such as target coverage (dose to 98% of the prescribed volume, *D*_98_), maximum dose (dose to 2% of the PTV, *D*_2_), MU, and DVH parameters related to OARs. To analyze the dosimetric effects of MSW on plan quality, conformity index (CI) and homogeneity index (HI) were calculated for each plan and defined as follows^20^, ^26^:

(1) $$Conformity\;Index\;=\frac{{\left(PTVpres\right)}^{2}}{\left(PTVvol\;\times VOLpres\right)},\;$$

where *PTVpres* represents the target volume encompassed by the prescription isodose, *PTVvol* represents the total target volume and *VOLpres* represents the total volume encompassed by the prescription dose.

(2) $$Homogeneity\;Index=\frac{\left(D2\%-D98\%\right)}{D50\%},$$

where *D*2% is the maximum dose received by 2% of the total target volume and *D*98% and *D*50% are the minimum dose received by 98% and 50% of the total target volume, respectively.

Lower HI represents good homogeneity; CI closer to 1 represents better conformality in dose distribution.^20^ MU efficiency calculated as the ratio between the number of MU for a plan and the ideal number of MU derived from the degree of modulation of the fluence profiles.^31^ Further, total treatment time and total number of segments created during optimization process, for each plan, was determined and compared for different MSWs.

### 2.2 Delivery analysis

All plans were delivered using Elekta Infinity® with Agility MLC® head (Elekta, Crawley, UK). The beam modulator head assembly consists of 80 leaf pairs (160 leaves in total) that are projected with a width of 5 mm at the isocenter. The accuracy of the dose delivery of all treatment plans with different MSW was measured using the Dolphin detector for all arc segments with the beam central axis oriented perpendicular to the plane of the detector. The effect of the MSW on delivery accuracy was quantitatively analyzed using the measured fluence with model‐based Compass dose verification system.

#### 2.2.1 Dolphin transmission detector

The transmission detector (Dolphin, IBA Dosimetry) is made up of pixel‐segmented ionization chambers, which is a series of 1513 air‐to‐air parallel plate chambers. The active measurement region of the detector is 24 cm × 24 cm whereas the diameter, height, and volume of the individual chambers were 3.2 mm, 2 mm, and 0.016 cc, respectively. The spatial resolution of the Dolphin detector is 5 mm for a field size of 14 cm × 14 cm and 10 mm outside this region. The Dolphin detector is mounted on the linear accelerator treatment head and is used to measure the fluence that linear accelerator produces for a given field. The measured fluence is used as input data to the 3D convolution algorithm, which allows the dose delivered by linear accelerator to be reconstructed in the CT data set that provides model‐based dose calculations and dose reconstruction based on 3D anatomy measurements.^24^, ^25^, ^32^

#### 2.2.2 Compass dose verification system

For each patient, treatment plans were exported from Monaco TPS to the Compass dose verification system as DICOM CT, RTSTRUCT, RTPLAN, and RTDOSE files that allow patient‐specific 3D dose reconstruction. Compass (v3.0) is a QA system with an internal beam model and a dose engine based on a collapsed cone convolution/superposition dose calculation algorithm that requires modeling of the linear accelerator head like any other TPS. Compass software consists of a detector model and a beam model that can predict the detector response through a response calculation algorithm. This estimated detector response is compared to the corresponding measured detector response. The differences obtained from the comparison results are provided as input to the final dose calculation.^11^, ^19^, ^21^, ^23^, ^33^, ^34^

#### 2.2.3 DVH and gamma analysis

Traditional global GI analysis was performed for all cases, both in calculations and measurements normalized to the maximum absolute dose of TPS. In all cases, a distance to agreement of 2 mm, a dose difference of 2%, and a lower dose threshold of 10% were used to exclude clinically irrelevant dose values. In all cases, a 95% percentage with a gamma value of 1 or less was used.^14^, ^35^

DVH information was gathered for the PTV and OARs of each group. The 3D pretreatment QA includes two parts: statistical evaluation of DVH analysis and GI analysis. The statistical evaluation compares the difference between the TPS computed and measurement reconstructed doses using three statistical parameters: the near‐maximum dose (*D*_1_—dose to 1% of the PTV), the average dose (*D*_mean_) and the minimum dose to 99% of the prescribed volume (*D*_99_) were calculated for PTVs, whereas the *D*_mean_ and *D*_1_ were studied for OARs. TPS dose constraints for OARs were also compared to the reconstructed dose measured with the Dolphin detector and the Compass verification method in each case. To increase the variability of cases, patients with different dose prescription were included in the study and analyzed in terms of percentages to compare them. 

### 2.3 Statistical analysis

The paired *t*‐test was applied in the intergroup comparison for dosimetric parameters and measurement results using the OriginPro software (Version 9.5, 2018).

## 3 RESULTS

Tables 1–4 illustrate the plan quality and deliverability parameters for each of the group compared. A total of 125 plans were made and compared across five groups.

**TABLE 1:** Brain

	Beam segment width (cm)				
Parameter	0.50 cm	0.80 cm	1.00 cm	1.25 cm	1.50 cm
**PTV (volume—290.6 cc ± 252.0 cc)/prescription dose—4825 cGy ± 1576 cGy**					
PTV V98%	94.9 ± 4.0	94.2 ± 4.9	94.4 ± 4.3	94.3 ± 4.5	91.6 ± 8.9
PTV *D*2%	105.6 ± 3.6	105.4 ± 4.0	106.2 ± 4.1	106.1 ± 4.5	106.3 ± 5.1
Conformity index (CI)	0.9 ± 0.1	0.9 ± 0.1	0.9 ± 0.1	0.9 ± 0.1	0.9 ± 0.1
Homogeneity index (HI)	0.1 ± 0.1	0.1 ± 0.1	0.1 ± 0.1	0.1 ± 0.1	0.1 ± 0.1
Monitor unit (MU)	699.9 ± 191.1	609.9 ± 235.5	560.4 ± 230.6	543.0 ± 232.6	537.1 ± 213.7
MU efficiency	72.8 ± 8.8	86.0 ± 9.9	91.5 ± 5.9	91.5 ± 5.8	94.3 ± 4.0
Estimated delivery time (s)	104.6 ± 31.2	100.5 ± 29.5	100.7 ± 28.5	101.8 ± 28.7	104.3 ± 29.0
Total number of segments	174.0 ± 27.5	159.3 ± 21.6	150.8 ± 18.3	143.8 ± 18.2	131.0 ± 28.9
**Plan deliverability (difference—TPS vs measured)**					
PTV					
*D*_mean_ (%)	1.1 ± 1.9	2.2 ± 0.8	2.0 ± 1.2	2.3 ± 0.7	2.3 ± 0.7
*D*_99_ (%)	1.5 ± 3.4	2.4 ± 2.2	2.4 ± 3.0	2.1 ± 3.1	2.0 ± 3.1
*D*_1_ (%)	0.6 ± 3.3	2.2 ± 1.0	2.3 ± 1.4	2.5 ± 1.1	2.8 ± 0.8
Brainstem (volume—32.5 cc ± 6.2 cc)					
*D*_mean_ (%)	3.7 ± 3.0	4.8 ± 5.9	5.9 ± 7.0	5.0 ± 7.3	6.1 ± 8.0
*D*_1_ (%)	0.4 ± 3.6	0.6 ± 3.8	1.3 ± 3.3	0.3 ± 3.3	2.2 ± 6.5
Optic chiasm (volume—1.6 cc ± 0.4 cc)					
*D*_mean_ (%)	1.3 ± 2.4	6.8 ± 13.2	6.6 ± 12.0	6.1 ± 13.1	4.9 ± 11.6
*D*_1_ (%)	1.1 ± 3.5	3.2 ± 10.9	3.8 ± 9.9	2.8 ± 9.9	2.3 ± 10.4
Optic nerve (volume—1.4 cc ± 0.6 cc)					
*D*_mean_ (%)	1.2 ± 4.2	1.5 ± 3.3	1.6 ± 3.9	1.8 ± 3.7	1.6 ± 3.8
*D*_1_ (%)	0.9 ± 3.7	2.2 ± 2.1	4.3 ± 6.7	2.6 ± 2.3	2.1 ± 2.7
Eye (volume—8.6 cc ± 1.0 cc)					
*D*_mean_ (%)	2.4 ± 10.6	5.4 ± 10.0	4.1 ± 8.9	4.5 ± 8.3	4.5 ± 9.3
*D*_1_ (%)	1.0 ± 9.9	2.4 ± 10.3	2.0 ± 12.0	1.0 ± 8.8	2.1 ± 9.4

**TABLE 2:** Head and neck

	Beam segment width (cm)				
Parameter	0.50 cm	0.80 cm	1.00 cm	1.25 cm	1.50 cm
**PTV (volume—207.1 cc ± 126.4 cc)/prescription dose—6162 cGy ± 1124 cGy**					
PTV V98%	97.2 ± 2.1	97.1 ± 2.1	95.9 ± 2.6	95.9 ± 2.4	95.4 ± 2.1
PTV *D*2%	107.6 ± 2.5	108.2 ± 2.9	108.6 ± 3.1	109.3 ± 4.5	110.3 ± 4.9
Conformity index (CI)	0.9 ± 0.2	0.9 ± 0.2	0.9 ± 0.2	0.9 ± 0.2	0.9 ± 0.2
Homogeneity index (HI)	0.2 ± 0.2	0.2 ± 0.2	0.2 ± 0.2	0.2 ± 0.2	0.2 ± 0.2
Monitor unit (MU)	1301.6 ± 363.3	1006.3 ± 140.3	877.4 ± 142.1	791.8 ± 111.8	784.0 ± 120.1
MU efficiency	69.4 ± 11.8	82.4 ± 11.2	92.0 ± 9.9	97.2 ± 4.8	98.2 ± 3.5
Estimated delivery time (s)	351.2 ± 37.6	320.1 ± 26.4	306.3 ± 21.1	288.3 ± 13.1	286.5 ± 12.4
Total number of segments	354.8 ± 20.6	321.8 ± 28.2	289.6 ± 25.9	267.8 ± 13.5	256.4 ± 14.0
**Plan deliverability (difference—TPS vs measured)**					
PTV					
*D*_mean_ (%)	0.07 ± 1.2	0.5 ± 1.1	0.5 ± 1.2	0.6 ± 1.6	2.0 ± 2.9
*D*_99_ (%)	1.2 ± 2.0	1.2 ± 1.9	1.2 ± 1.9	1.2 ± 1.7	2.7 ± 5.6
*D*_1_ (%)	0.5 ± 2.1	0.5 ± 1.3	0.6 ± 1.5	0.9 ± 1.8	1.3 ± 6.2
Spinal cord (volume—12.4 cc ± 2.9 cc)					
*D*_mean_ (%)	2.9 ± 2.4	3.2 ± 2.4	2.9 ± 2.3	3.3 ± 1.9	4.3 ± 3.3
*D*_1_ (%)	1.4 ± 2.7	1.6 ± 3.1	1.4 ± 2.8	1.6 ± 2.6	3.5 ± 6.3
Parotid (volume—33.6 cc ± 12.4 cc)					
*D*_mean_ (%)	3.5 ± 1.7	4.2 ± 1.8	4.2 ± 1.9	4.4 ± 2.8	4.1 ± 3.2
*D*_1_ (%)	1.2 ± 1.8	1.6 ± 1.8	1.4 ± 1.6	1.4 ± 1.6	1.4 ± 1.8
Oral cavity (volume—78.8 cc ± 20.9 cc)					
*D*_mean_ (%)	1.1 ± 1.4	1.6 ± 2.1	1.3 ± 1.6	2.1 ± 1.4	2.1 ± 1.5
*D*_1_ (%)	0.5 ± 0.8	0.7 ± 1.8	0.2 ± 1.6	0.5 ± 2.1	0.5 ± 1.6
Mandible (volume—74.0 cc ± 10.0 cc)					
*D*_mean_ (%)	3.8 ± 1.1	3.4 ± 1.8	3.4 ± 1.0	3.9 ± 2.2	2.9 ± 3.0
*D*_1_ (%)	1.1 ± 1.6	1.3 ± 1.2	0.8 ± 1.0	0.9 ± 1.7	3.3 ± 5.5
Larynx (volume—18.7 cc ± 1.9 cc)					
*D*_mean_ (%)	1.5 ± 0.2	1.8 ± 0.3	1.6 ± 0.6	1.9 ± 0.4	1.7 ± 0.2
*D*_1_ (%)	0.9 ± 0.1	1.2 ± 0.1	0.8 ± 0.1	0.8 ± 0.6	0.9 ± 0.4
Esophagus (volume—9.2 cc ± 2.1 cc)					
*D*_mean_ (%)	2.1 ± 0.7	1.7 ± 0.8	1.5 ± 0.8	2.2 ± 0.9	4.0 ± 2.7
*D*_1_ (%)	0.6 ± 1.8	1.6 ± 1.7	1.4 ± 1.1	0.5 ± 1.9	1.5 ± 6.0

**TABLE 3:** Thorax

	Beam segment width (cm)				
Parameter	0.50 cm	0.80 cm	1.00 cm	1.25 cm	1.50 cm
**PTV (volume—153.2 cc ± 129.0 cc)/prescription dose—5441.25 cGy ± 423 cGy**					
PTV V98%	94.9 ± 3.8	94.2 ± 4.2	95.6 ± 4.5	90.1 ± 8.0	90.1 ± 6.9
PTV *D*2%	107.1 ± 1.5	107.6 ± 1.8	107.7 ± 1.6	107.9 ± 1.5	107.6 ± 1.6
Conformity index (CI)	0.9 ± 0.1	0.9 ± 0.1	0.9 ± 0.1	0.9 ± 0.1	0.9 ± 0.1
Homogeneity index (HI)	0.1 ± 0.1	0.1 ± 0.1	0.1 ± 0.1	0.1 ± 0.1	0.1 ± 0.1
Monitor unit (MU)	1490.5 ± 741.2	1029.7 ± 331.9	976.7 ± 359.4	920.6 ± 266.1	967.5 ± 331.6
MU efficiency	82.8 ± 8.1	97.0 ± 4.2	97.5 ± 5.0	99.0 ± 2.0	98.5 ± 3.0
Estimated delivery time (s)	214.6 ± 65.8	191.3 ± 53.3	192.2 ± 56.3	191.0 ± 52.7	198.2 ± 55.0
Total number of segments	303.0 ± 55.3	258.0 ± 32.3	229.3 ± 31.6	217.8 ± 18.0	206.8 ± 17.7
**Plan deliverability (difference—TPS vs measured)**					
PTV					
*D*_mean_ (%)	0.4 ± 1.7	0.6 ± 1.1	2.9 ± 2.2	1.7 ± 1.7	1.3 ± 0.8
*D*_99_ (%)	0.3 ± 3.3	1.7 ± 5.9	5.9 ± 7.0	1.7 ± 2.7	0.4 ± 4.6
*D*_1_ (%)	0.5 ± 2.3	1.5 ± 0.8	2.0 ± 1.0	2.6 ± 1.4	2.3 ± 0.4
Ipsilateral lung (volume—1394 cc ± 463 cc)					
*D*_mean_ (%)	0.7 ± 1.9	2.3 ± 3.9	2.8 ± 4.1	2.0 ± 3.0	2.5 ± 2.5
*D*_1_ (%)	0.9 ± 1.2	1.3 ± 0.7	1.9 ± 1.4	1.9 ± 1.9	1.9 ± 0.5
Contralateral lung (volume—1256 cc ± 220 cc)					
*D*_mean_ (%)	2.5 ± 3.1	2.1 ± 4.3	2.5 ± 7.1	5.2 ± 4.9	4.4 ± 8.9
*D*_1_ (%)	2.6 ± 4.7	2.8 ± 5.1	5.9 ± 10.0	0.4 ± 10.8	4.0 ± 11.1
Heart (volume—549 cc ± 110 cc)					
*D*_mean_ (%)	2.8 ± 3.6	3.1 ± 3.7	3.7 ± 1.9	3.2 ± 2.7	2.5 ± 2.0
*D*_1_ (%)	0.3 ± 2.3	0.4 ± 2.5	1.4 ± 3.1	0.6 ± 2.8	0.9 ± 2.7

**TABLE 4:** Pelvis and extremity

	Beam segment width (cm)				
Parameter	0.50 cm	0.80 cm	1.00 cm	1.25 cm	1.50 cm
**Pelvis—PTV (volume—428.5 cc ± 492.0 cc)/prescription dose—4985 cGy ± 1773 cGy**					
**Extremity—PTV (volume—298.6 cc ± 505.4 cc)/prescription dose—3875 cGy ± 1493 cGy**					
PTV V98% (pelvis)	94.5 ± 4.7	94.0 ± 5.3	93.6 ± 5.8	93.1 ± 5.5	91.7 ± 7.1
PTV V98% (extremity)	99.7 ± 0.3	99.5 ± 0.8	97.9 ± 4.0	97.8 ± 3.8	95.8 ± 7.4
PTV *D*2% (pelvis)	108.9 ± 5.8	109.9 ± 6.2	109.6 ± 6.1	109.9 ± 6.2	110.5 ± 7.4
PTV *D*2% (extremity)	109.5 ± 5.0	109.9 ± 5.9	110.4 ± 6.8	110.3 ± 6.8	110.8 ± 7.3
Conformity index (pelvis)	1.0 ± 0.0	1.0 ± 0.0	1.0 ± 0.1	1.0 ± 0.1	1.0 ± 0.2
Conformity index (extremity)	0.9 ± 0.1	0.9 ± 0.1	0.9 ± 0.1	0.9 ± 0.1	0.9 ± 0.1
Homogeneity index (pelvis)	0.1 ± 0.1	0.1 ± 0.1	0.1 ± 0.1	0.1 ± 0.1	0.1 ± 0.1
Homogeneity index (extremity)	0.1 ± 0.1	0.2 ± 0.1	0.2 ± 0.1	0.2 ± 0.1	0.2 ± 0.1
Monitor unit (pelvis)	1657.6 ± 475.6	1325.9 ± 459.0	1266.7 ± 438.2	1192.9 ± 441.5	1183.3 ± 485.3
Monitor unit (extremity)	1144.0 ± 476.0	991.0 ± 459.2	917.2 ± 438.8	889.3 ± 441.2	898.5 ± 485.0
MU efficiency (pelvis)	60.1 ± 19.1	74.1 ± 21.2	77.0 ± 21.0	80.0 ± 22.3	80.9 ± 22.9
MU efficiency (extremity)	75.5 ± 15.0	87.0 ± 12.0	93.5 ± 11.1	95.5 ± 9.0	97.8 ± 4.5
Estimated delivery time (s—pelvis)	284.5 ± 107.3	261.1 ± 88.6	254.5 ± 78.6	254.6 ± 82.4	252.8 ± 77.5
Estimated delivery time (s—extremity)	123.3 ± 38.5	117.1 ± 43.3	115.3 ± 43.3	117.0 ± 47.0	123.7 ± 53.6
Total number of segments (pelvis)	308.7 ± 68.9	281.3 ± 69.1	258.0 ± 58.0	260.9 ± 71.9	247.4 ± 68.3
Total number of segments (extremity)	303.0 ± 100.9	237.3 ± 107.7	223.0 ± 110.0	215.3 ± 91.7	199.3 ± 78.1
**Plan deliverability (difference—TPS vs measured)**					
PTV					
*D*_mean_ (%)	0.2 ± 1.0	0.1 ± 0.8	0.4 ± 1.7	0.4 ± 0.7	0.3 ± 0.9
*D*_99_ (%)	0.4 ± 1.7	0.5 ± 0.9	0.3 ± 1.7	0.7 ± 1.2	0.6 ± 1.7
*D*_1_ (%)	0.4 ± 1.0	0.6 ± 1.0	1.3 ± 1.4	1.1 ± 1.1	0.5 ± 1.0
Liver (volume—1367.0 cc ± 359.0 cc)					
*D*_mean_ (%)	4.3 ± 2.3	7.5 ± 6.7	6.0 ± 4.9	4.9 ± 2.9	6.8 ± 5.7
*D*_1_ (%)	0.5 ± 1.6	3.9 ± 7.6	3.8 ± 7.2	2.5 ± 2.0	2.5 ± 4.0
Kidney (volume—179.0 cc ± 26.0 cc)					
*D*_mean_ (%)	2.2 ± 2.5	2.3 ± 2.5	2.2 ± 2.0	2.6 ± 1.1	2.7 ± 1.1
*D*_1_ (%)	0.5 ± 1.4	0.8 ± 1.6	0.4 ± 1.0	0.7 ± 1.2	1.1 ± 1.3
Bladder (volume—306.0 cc ± 276.0 cc)					
*D*_mean_ (%)	1.6 ± 1.9	1.7 ± 1.9	2.3 ± 3.1	1.9 ± 3.0	2.5 ± 2.5
*D*_1_ (%)	0.4 ± 1.2	0.3 ± 0.7	0.5 ± 1.4	1.1 ± 1.9	0.5 ± 1.5
Rectum (volume—40.6 cc ± 15.6 cc)					
*D*_mean_ (%)	1.9 ± 1.8	1.5 ± 1.8	2.8 ± 1.9	1.4 ± 2.8	1.4 ± 3.2
*D*_1_ (%)	1.6 ± 1.5	1.6 ± 1.8	1.8 ± 1.6	1.4 ± 1.6	1.4 ± 1.8
Femoral head (combined) (volume—92.3 cc ± 12.3 cc)					
*D*_mean_ (%)	4.8 ± 2.0	4.6 ± 2.1	6.2 ± 3.2	5.7 ± 2.9	6.8 ± 3.1
*D*_1_ (%)	3.6 ± 1.2	3.8 ± 1.6	4.6 ± 2.0	3.8 ± 2.1	5.4 ± 3.2

### 3.1 Treatment plan quality

When compared to plans using other MSWs, the target dose coverage of the plans using MSW of 0.5 cm was found to be superior for all groups. PTV coverage (*D*_98_) decreased significantly (*p* < 0.05) as the MSW increased. Similarly, the maximum dose (*D*_2_) was found to be increased significantly (*p* < 0.05) as the MSW increased, with MSW of 1.5 cm being the least in terms of plan quality for both PTVs and OARs. A CI of >0.9 and above was achieved for all calculated plans. The plans created using MSW of 0.5 cm provided superior CI. Similarly, HI of <0.2 was achieved for all plans with MSW of 0.5 cm provided lowest HI.

**FIGURE 1:**
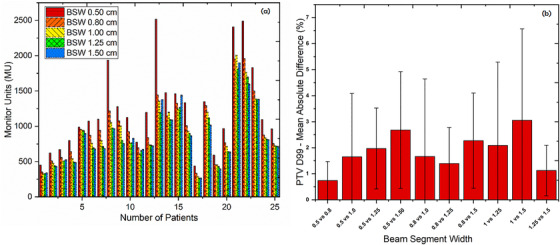
(a) Monitor units for all plans using different minimum segment width. (b) Mean absolute difference in percentage for *D*_99_ (PTV) in TPS calculated treatment plans between different minimum segment width

The VMAT plan's MUs decreased as the MSW increased (Figure 1a); the mean MUs for plans with MSWs of 0.5, 0.8, 1.0, 1.25, and 1.5 cm, as well as plan quality parameters, are reported in Tables 1–4, respectively. The plan with 0.5 cm MSW had a higher total MU value in all cases. Similarly, MU efficiency increased as the MSW increased but no correlation was found with plan quality. As shown in Tables 1–4, the total number of segments created during optimization process was significantly higher for MSW of 0.5 cm as compared to other MSWs (*p* < 0.05). Also, in terms of total treatment time, only head and neck plans created using MSW of 0.5 cm was significantly higher as compared to other MSWs (*p* < 0.05). Further, no significant correlation was found between monitor units and PTV volumes (*r* ^2^ = 0.31). Figure 1b illustrates the mean difference between MSWs for *D*_99_ of the target volume.

**FIGURE 2:**
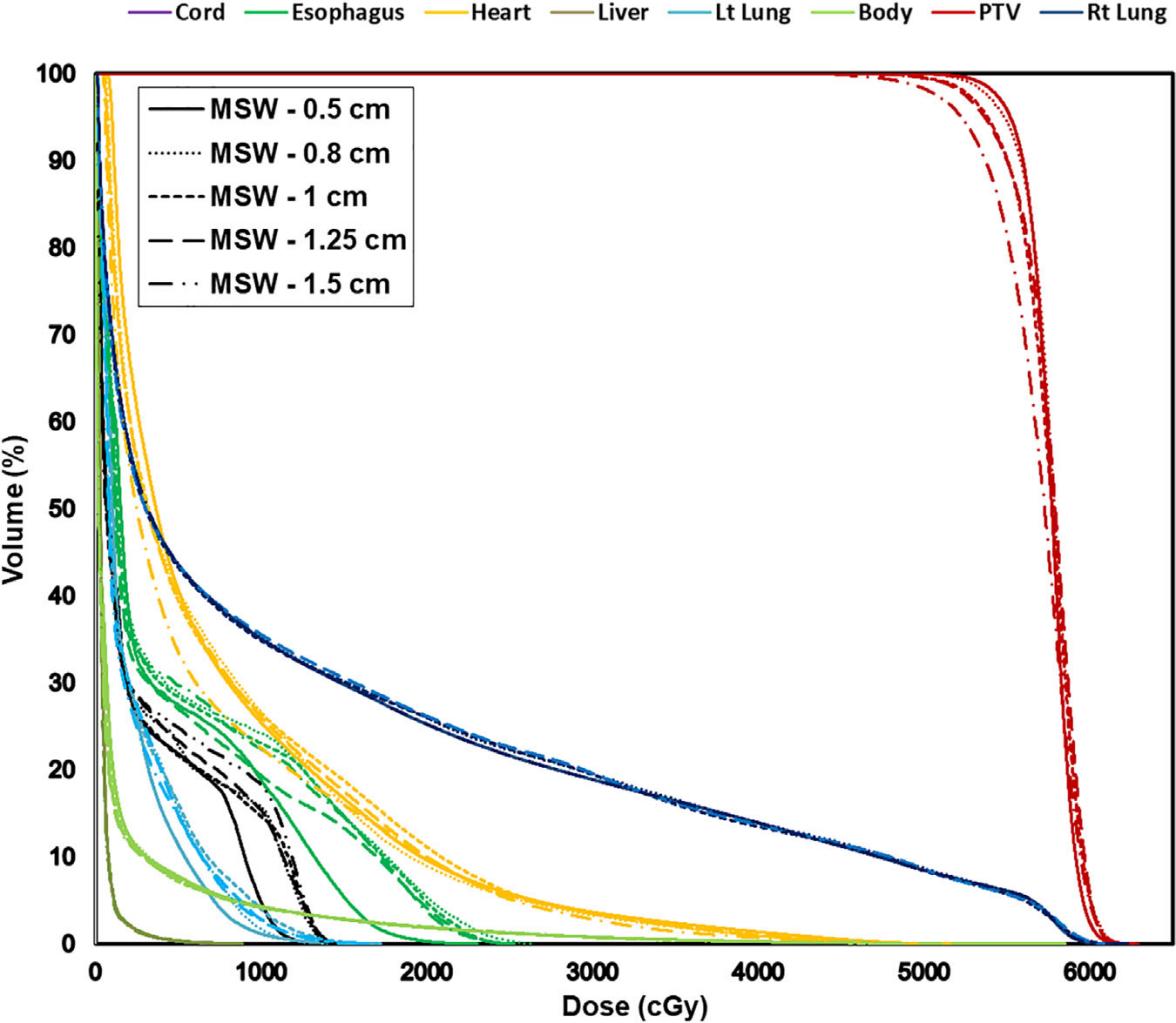
The DVH comparison of a volumetric modulated arc therapy plans for a Lung case created using different minimum segment widths (MSWs)

To ensure a fair comparison of OARs, the plans with 0.5 cm of MSW were used as a baseline and compared to the other four plans in each case. Except for OARs with small volumes (Lens, Optic nerves, Cochlea), the *D*_mean_ for OARs using different MSW as compared to 0.5 cm was within ±5% of each other in all groups. However, for *D*_1_ (maximum dose) to the spinal cord, ipsilateral lung, heart, brainstem, and esophagus, there was a difference of up to 14%, 20%, 12%, 10%, and 22%. As the MSW increased sequentially, the dose constraint for some OARs exceeded the tolerance limits, particularly in brain and head and neck groups, when compared to MSW of 0.5 cm. Figure 2 illustrates a DVH comparison of a typical lung case planned using different MSWs.

### 3.2 Treatment plan deliverability

The average percentage of passed gamma values achieved was above 95% for all cases (MSW 0.5 cm—99.5% ± 0.8%, MSW 0.8 cm—99.3% ± 1.1%, MSW 1.0 cm—99.4% ± 1.1%, MSW 1.25 cm—99.5% ± 1.0%, and MSW 1.50 cm—99.5% ± 0.7%) using 2% 2‐mm gamma criteria, respectively. However, no correlation was observed between gamma passing rates and DVH difference (%) for the target volumes (*r* ^2^ = 0.21).

Tables 1–4 summarize the quantitative comparison for PTVs (*D*_99_, *D*_mean_, and *D*_1_) based on DVH analysis of dose distributions calculated with Monaco TPS and reconstructed doses using measured fluence computed with Dolphin/Compass. Data are presented separately for each group, with the mean over all patients in the group and the standard deviation for each parameter. With TPS computed to measured fluence, VMAT plans produced with MSW of 0.5 cm showed a better dosimetric index and a smaller deviation. The quality and deliverability of the plans deteriorated as the MSW increased.

**FIGURE 3:**
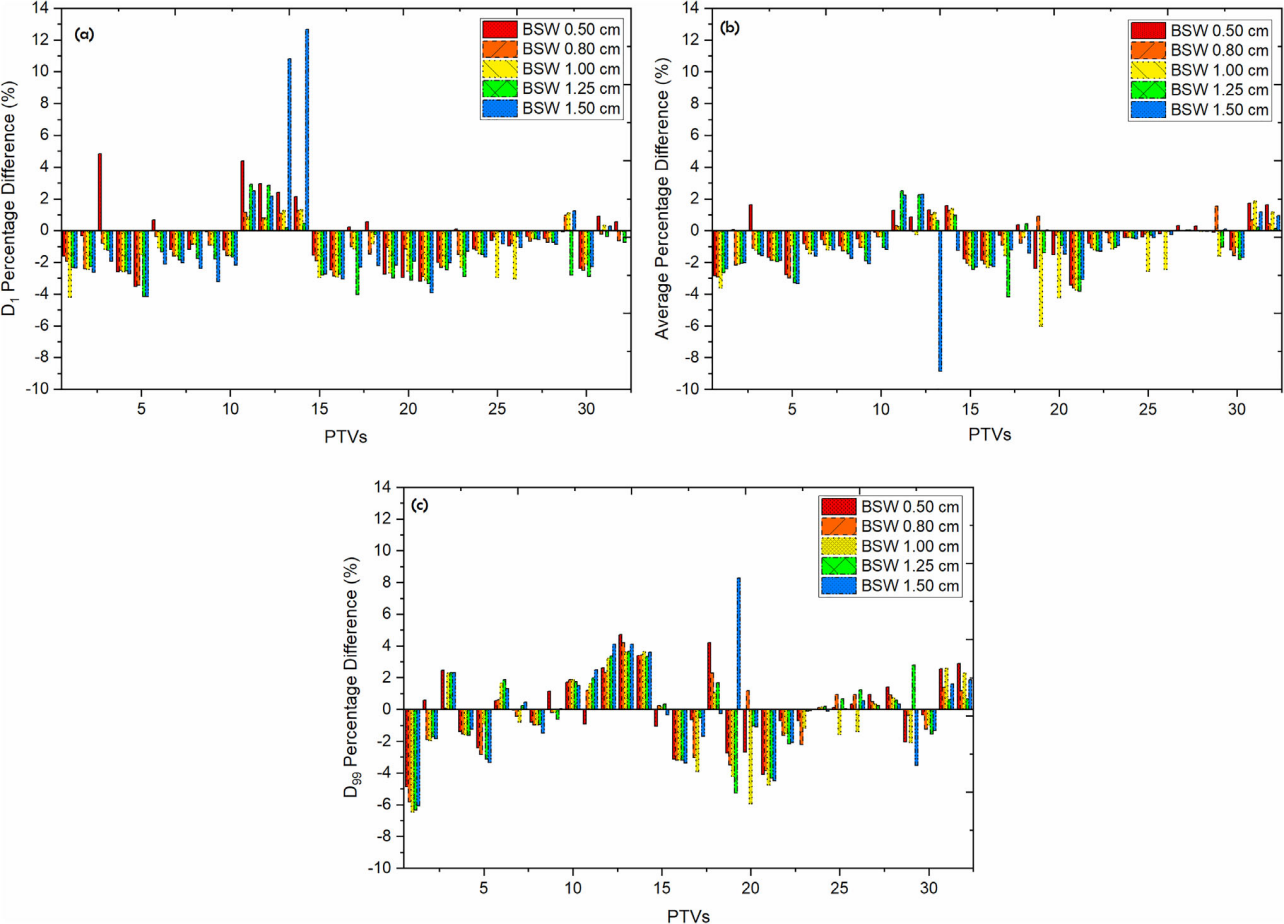
Average percentage difference between the TPS calculated plan and measured fluence using DVH analysis for *D*_1_ (a), *D*_mean_ (b), and *D*_99_ (c)

For various MSWs, Figure 3 shows the percentage difference between TPS calculated and reconstructed dose for PTVs using DVH analysis. The result demonstrates that as the MSW increases, the difference between the computed and measured fluence increases. For PTV parameters (*D*_1_, *D*_mean_, and *D*_99_), the maximum absolute difference was observed to be 8%, 13%, and 8%, respectively. TPS plans produced with MSW of 0.5 cm correlated well with measured reconstructed dose for target volumes and OARs, as shown in Tables 1–4, when compared to other MSWs. Similarly, for all sites, the reconstructed dose for individual OARs correlated well with the TPS planned dose, using MSW 0.5 cm as compared to other MSWs. Furthermore, as shown in Tables 1–4, large differences in OAR doses using MSW other than 0.5 cm were observed, particularly in cases involving the brain and thorax.

## 4 DISCUSSION

The choice of the optimization and segmentation parameters is essential to obtain the best compromise between the quality of the dosimetry and irradiation parameters. The sequencing parameters would affect the quality and deliverability of a treatment plan just as much as the constraints will. This study used DVH analysis with a transmission‐based detector (Dolphin) and a model‐based Compass verification system to determine the optimal MSW for Monte Carlo‐based clinical treatment planning for various sites. With respect to various MSWs, the study looked at the relationship between MSW and clinical planning quality/deliverability. The study compared tumor coverage, CI, and HI to determine the plan quality of various plans. Similarly, the plan's deliverability was also assessed using the Dolphin/Compass dose verification system. This is the first study to assess plan quality and deliverability across a wide range of clinical settings using a variety of MSWs utilizing DVH analysis.

Previous studies have identified the effects of MSW on the quality of VMAT plans for multiple VMAT cases in clinical treatment.^8^, ^26^, ^27^, ^28^ Wang et al. and Nithyanantham et al. reported the influence of MSW on the quality and delivery accuracy of VMAT plans for cervix cancer and stereotactic body radiotherapy.^8^, ^26^ The studies demonstrated a similar result to the present study with regards to reduced MU and treatment time with increased MSW. However, in contrast, Wang et al. reported no significant difference in target dose coverage with MSW of 1.0 cm and 1.50 cm as compared to MSW of 0.5 cm. This could be the result of other factors such as the fluence smoothing and grid size chosen between studied which could influence the dose coverage. Previous studies were limited to a small range of MSWs, and plan deliverability was assessed solely through GI analysis, with no clinical correlation.

We evaluated a total of 125 treatment plans using VMAT treatment planning technique. The TPS plans were compared to their Dolphin‐measured and Compass‐computed counterparts. The HI, CI, tumor coverage, maximum doses, and mean doses to the PTV, as well as the dosage volume index of the OARs and MUs, were all evaluated as part of the treatment plan quality comparisons. The quality and deliverability of plans deteriorated as the MSW increased, according to the study. The MU, on the other hand, was found to be significantly higher for MSW of 0.5 cm and gradually decreased as the MSW increased. An increase in total treatment time and reduced MU efficiency was observed for MSW of 0.5 cm in this study because it is self‐evident that a bigger MU would result in a longer treatment time and lower MU efficiency. However, it has been demonstrated that MU efficiency does not correlate with plan quality and deliverability. As shown in Tables 1–4, a higher MU efficiency achieved for a MSW of 1.5 cm in all cases evaluated which had the lowest plan quality. A smaller MSW would results in large number of segments with finer grid size of the fluence map during optimization, which would leads to better quality plans.

Complexity has previously been defined in terms of a VMAT treatment plan as “the frequency and amplitude of fluctuations in the intensity distribution of a beam,” while others have defined increasing complexity simply by the number of monitor units. A high degree of complexity is not always a negative feature of a treatment plan, as it may be needed due to the geometry and location of the target and organs at risk, and there is a tradeoff between complexity and treatment quality in terms of meeting planning goals.^36^

Because dose calculation in VMAT treatment plans is more complicated, verification of treatment delivery and comparison of TPS calculated dose versus measured dose are critical. Although the 2%/2 mm GI analysis with a passing rate greater than 95% was used in this study, several studies have shown that it is unreliable for treatment plan acceptability.^17^, ^34^ Despite being calculated in full density, the patient GI passing rate for the entire dose grid has a poor correlation to errors in the DVH‐based metrics, according to the study. If the general GI passing rate is thought of as a nonpatient specific metric, the lack of correlation in patient‐specific QA is understandable. In terms of radiotherapy treatment, not all voxels in a patient's image are created equal. If dose errors coincide geometrically with critical structures, such as target volumes or OARs, they can have a significant clinical impact, whereas if they occur outside of critical structures, they can have a minor clinical impact. Critical volumes come in a variety of shapes, sizes, and locations, depending on the patient. Even for the real patient anatomy, the GI passing rate for the entire dose volume does not provide information about the anatomical location of the failure or the dose level at which it occurred, both of which are critical. Although the GI passing rate provides information on the number of errors, it does not provide information on the magnitude of the errors. Due to the lack of correlation between global GI pass rate and dose difference in target volumes and OARs, DVH metrics‐based QA for VMAT has been proposed, which compares directly the TPS calculated and measured 3D dose distribution.^11^, ^16^, ^17^, ^37^, ^38^

In general, the Dolphin/Compass DVH‐based dose assessment provides a comparative analysis between TPS planned and reconstructed dose distribution for targets and OARs for all sites, allowing for a better interpretation of clinical effect. Because of its ability to calculate 3D dose on a patient CT scan using beam modeling, array detector measurement, and treatment plan, Dolphin/Compass dosimetry system outperforms many other QA systems. When compared to TPS using the Compass dose verification system and measured fluence, VMAT plans with 0.5 cm of MSW had a higher dosimetric index and lower deviation. The plan with 1.5 cm of MSW, on the other hand, had a lower plan quality and a higher deviation in deliverability when TPS calculated fluence verification was used.

The dose distribution measured using Dolphin detector mimics the dose distribution within the patient, distorted and changed only by the difference between the patient and a lack of heterogeneities in the phantom, because the recorded doses from the Dolphin detector are from all the beams in the plan at their intended positions. Uniform high‐dose regions will be present within the detector array, along with comparable dose gradients and low‐dose regions found in the patient's plan. The Dolphin detector and Compass dose verification system has the advantage of measuring gantry, collimator, and MLC leaf position inaccuracies with gantry angle as well as accurate data transfer. Furthermore, the resulting planar dose distribution is closely related to the dose that will be given to the patient, allowing for the assessment of the relationship between the high‐dose area and organs at risk lying in the same plane.^10^

Despite the fact that each of the 1513 air‐vented chambers in the Dolphin detector had a diameter of 3.2 mm, the Compass system was able to accurately reconstruct dose distributions for small fields due to a reconstruction technique that combines measured fluence with beam modeling. The Compass system was sensitive enough to detect a change in dose at the detector and then reconstruct the dose distribution from it. Godart et al. also demonstrated the Compass system's capability to detect MLC leaf position errors.^21^ Because most leaf position errors are less than a millimeter, detecting the MLC error requires a high‐resolution detector. However, the reconstructed dose for measurement‐based QA is composed using perturbative corrections based on signals measured with the Dolphin detector array. Over the entire computed fluence, the differences between predicted and measured fluence are corrected with a delivery scaling factor, and residual response corrections in selected regions are handled element by element. When the total integrated chamber response is known and greater resolution is desired, Monte Carlo (MC)‐based modeling is used to resolve the array's inherent low resolution on a pixel‐by‐pixel basis.^24^

## 5 LIMITATIONS

When compared to QA systems like film dosimetry, the accuracy of ionization detectors is subject to uncertainties due to volume averaging, geometrical resolution, and self‐attenuation, which raises concerns about their sensitivity. Furthermore, since the Dolphin detector is mounted on the linear accelerator's head, errors such as table rotation are not detectable. In Compass dose verification system, a Monte Carlo generated response function for each ion chamber is used to overcome the detector resolution restriction. However, the dose calculation algorithm employed in Compass dose verification system is Collapsed Cone algorithm as compared to Monte Carlo algorithm in TPS. Furthermore, this research does not include online measurements with the Dolphin detector. Besides, the research is limited to a single institution's treatment planning and delivery system.

## 6 CONCLUSION

DVH analysis demonstrated that treatment plans with minimal MSW showed better plan quality and deliverability and provided clinical relevance as compared to GI analysis. In an overall view among the compared MSW, the minimum segment width of 0.5 cm represented a clear merit in plan quality and deliverability, in terms of PTV and OARs for all VMAT treatment plans.

## CONFLICT OF INTEREST

There are no conflicts of interest.

## ETHICS STATEMENT

No ethics approval was required for this study. No copyright material has been used that requires approval for it to be published in this manuscript.

## AUTHOR CONTRIBUTIONS

Study conception and design: all authors. Data collection: AB Mohamed Yoosuf, MB Ahmad, and M Alqathami. Analysis and interpretation of results: AB Mohamed Yoosuf, MB Ahmad, M Alqathami, S Shehri, and A Alhadab. Draft manuscript preparation: AB Mohamed Yoosuf, MB Ahmad, M Alqathami, S Shehri, and A Alhadab. All authors reviewed the results and approved the final version of the manuscript.
